# Walking Speed Reliably Measures Clinically Significant Changes in Gait by Directional Deep Brain Stimulation

**DOI:** 10.3389/fnhum.2020.618366

**Published:** 2021-01-29

**Authors:** Christopher P. Hurt, Daniel J. Kuhman, Barton L. Guthrie, Carla R. Lima, Melissa Wade, Harrison C. Walker

**Affiliations:** ^1^Rehabilitation Sciences, University of Alabama at Birmingham, Birmingham, AL, United States; ^2^Department of Physical Therapy, University of Alabama at Birmingham, Birmingham, AL, United States; ^3^Department of Neurosurgery, University of Alabama at Birmingham, Birmingham, AL, United States; ^4^Department of Neurology, University of Alabama at Birmingham, Birmingham, AL, United States; ^5^Department of Biomedical Engineering, University of Alabama at Birmingham, Birmingham, AL, United States

**Keywords:** Parkinson's disease, deep brain stimulation, walking speed, reliability, step length

## Abstract

**Introduction:** Although deep brain stimulation (DBS) often improves levodopa-responsive gait symptoms, robust therapies for gait dysfunction from Parkinson's disease (PD) remain a major unmet need. Walking speed could represent a simple, integrated tool to assess DBS efficacy but is often not examined systematically or quantitatively during DBS programming. Here we investigate the reliability and functional significance of changes in gait by directional DBS in the subthalamic nucleus.

**Methods:** Nineteen patients underwent unilateral subthalamic nucleus DBS surgery with an eight-contact directional lead (1-3-3-1 configuration) in the most severely affected hemisphere. They arrived off dopaminergic medications >12 h preoperatively and for device activation 1 month after surgery. We measured a comfortable walking speed using an instrumented walkway with DBS off and at each of 10 stimulation configurations (six directional contacts, two virtual rings, and two circular rings) at the midpoint of the therapeutic window. Repeated measures of ANOVA contrasted preoperative vs. maximum and minimum walking speeds across DBS configurations during device activation. Intraclass correlation coefficients examined walking speed reliability across the four trials within each DBS configuration. We also investigated whether changes in walking speed related to modification of step length vs. cadence with a one-sample *t*-test.

**Results:** Mean comfortable walking speed improved significantly with DBS on vs. both DBS off and minimum speeds with DBS on (*p* < 0.001, respectively). Pairwise comparisons showed no significant difference between DBS off and minimum comfortable walking speed with DBS on (*p* = 1.000). Intraclass correlations were ≥0.949 within each condition. Changes in comfortable walk speed were conferred primarily by changes in step length (*p* < 0.004).

**Conclusion:** Acute assessment of walking speed is a reliable, clinically meaningful measure of gait function during DBS activation. Directional and circular unilateral subthalamic DBS in appropriate configurations elicit acute and clinically significant improvements in gait dysfunction related to PD. Next-generation directional DBS technologies have significant potential to enhance gait by individually tailoring stimulation parameters to optimize efficacy.

## Introduction

Deep brain stimulation (DBS) is an effective therapy for Parkinson's disease (PD) patients in whom pharmacologic interventions no longer optimally control motor symptoms (Walker et al., 2009). Addressing functional declines in gait and balance with disease progression are major unmet needs in PD therapeutics. Although bilateral DBS often improves gait function, outcomes vary, and relatively little is known about changes in gait with both unilateral surgery and directional lead technologies.

Traditional ring-shaped DBS electrodes deliver a symmetrical electrical field in an open loop manner. Newer technologies incorporate novel lead designs with radially oriented (directional) contact segments and closed-loop sensing. While these advances promise to improve efficacy and minimize side effects, they also increase the complexity of device activation. Routine DBS programming sessions, even without incorporating new technologies, are challenged by subjective elements of motor assessments, order effects, and the questionable reliability of repeated behavioral measurements in individuals. Quantitative, reliable, and simple measures are therefore needed to translate potential advantages of these emerging DBS technologies into improved functional outcomes.

Gait is a vital, integrated motor behavior that incorporates elements of appendicular function, posture, and balance. Standard PD rating scales measure gait in a relatively insensitive manner, and typical clinical DBS programming sessions do not systematically measure changes gait speed across each of the available contacts on the lead (Inzitari et al., 2017). Comfortable walking speed (CWS) is a validated measure that could serve as a simple clinical tool to assess DBS efficacy for gait function, and potentially as a proxy for balance, posture, and other motor symptoms. Slower walk speed is a robust finding in PD relative to healthy controls (Morris et al., 1996; Kuhman et al., 2018). Gait deficits in PD are primarily caused by decreased step length with preservation of cadence (Morris et al., 1994, 2001). While step length is often responsive to levodopa and DBS (Navratilova et al., 2020; Peterson et al., 2020), PD patients disproportionately increase cadence when voluntarily increasing walk speed (Morris et al., 1996; Peterson et al., 2020).

A previous metanalysis showed that DBS improves walking speed; however, outcomes varied substantially from large to negligible improvements (Roper et al., 2016), and progressive declines in gait and balance remain major unmet needs in PD therapeutics. DBS programming practices vary substantially worldwide, and systematically addressing gait dysfunction often may not be an explicit behavioral goal. The extent to which an initial DBS programming session generates a meaningful range of walk speeds across electrode contacts is unknown. Here we investigate whether DBS changes walking speed reliably and to a clinically meaningful extent during initial programming with both directional and circular stimulation. We also quantified the extent to which changes in walk speed resulted from changes in the scaling of step length vs. increased cadence.

## Materials and Methods

We tested 19 PD patients (Table 1) who underwent unilateral subthalamic nucleus (STN) DBS as part of a larger clinical trial (National Institute of Health BRAIN Initiative, clinicaltrials.gov NCT03353688). This protocol was approved by the Institutional Review Board of the University of Alabama at Birmingham. All participants provided written informed consent prior to participation, only after a multidisciplinary committee recommended DBS at the STN target as part of routine care. Inclusion in the study required ≥30% improvement in the MDS-UPDRS part III after self-administered dopaminergic medication compared to their off state (≥12 off dopaminergic medication) assessed during a pre-operative screening visit (pre-op) in which we also assessed CWS. Other inclusion criteria included ages 18–70 years old, Hoehn and Yahr classification >1 (4 maximum), and a Dementia Rating Scale-2 score ≥ 130 (out of 144). Exclusion criteria included duration of PD <4 years, history of stroke or other neurological conditions (i.e., history of seizures), and diagnosis of psychogenic movement disorder based on consensus criteria. For those who qualified for the study, we implanted a directional DBS lead (Boston Scientific Vercise DBS system, Natick MA, USA) in the STN contralateral to the most affected side of the body.

**Table 1:** Demographic and clinical features of enrolled participants.

**Subject**	**Age**	**Years since diagnosis**	**Stage of disease**	**PIGD Score**	**Falls in past year**	**Multiple falls in past year**	**FOG-Q**	**OFF**	**ON**	**DRS-2**
1	47	7	2	0.8	Yes	No	7	73	29	132
2	62	9	2	1.0	Yes	Yes	2	52	25	139
3	63	5	3	0.8	No	No	2	45	26	134
4	56	7	2	0.2	No	No	0	42	29	141
5	59	11	2	1.6	No	No	8	53	30	138
6	62	8	3	2.2	Yes	Yes	11	80	41	133
7	63	5	2	0.2	No	No	0	33	17	141
8	45	8	2	1.8	Yes	Yes	13	44	14	142
9	62	4	2	1.0	No	No	11	53	35	136
10	66	8	2	1.2	No	No	15	46	37	139
11	70	4	2	0.8	Yes	Yes	10	59	29	135
12	63	12	2	0.8	Yes	Yes	6	53	36	140
13	54	12	2	0.8	No	No	8	55	27	138
14	63	10	2	1.0	No	No	0	47	31	139
15	57	6	2	0.4	No	No	0	17	9	133
16	54	5	3	0.8	Yes	Yes	2	47	32	136
17	51	4	2	0.2	Yes	Yes	14	33	18	143
18	49	3	3	1.6	No	No	1	77	39	137
19	59	8	2	0.6	Yes	Yes	12	53	25	141
Mean	58.2	7.2	2.2	0.9	9 Fallers	8 Multi-fallers	6.4	51.2	27.8	137.7
St. dev.	6.7	2.8	0.4	0.5			5.4	15.1	8.6	3.3

*Stage of Disease, Hoehn and Yahr; FOG-Q, Freezing of Gait Questionnaire; OFF and ON, MDS-UPDRS III; DRS-2, Dementia Rating Scale-2; PIGD, Postural Instability and Gait Disability*.

### Monopolar Survey

Approximately 4 weeks after the implant, participants arrived in the morning in the practically defined off state (≥12 h after last administration of PD medicines) for a monopolar review. Participants were advised to forgo their nightly dose of any extended-release medications. An experienced, certified movement disorders clinician conducted DBS programming using monopolar configuration and standard pulse width of 60 μs and a frequency of 130 Hz in all cases. Participants and researchers were blinded to DBS settings at all times except the movement disorders clinician. The directional DBS lead contains four rows, consisting of eight total contacts in a “1-3-3-1” configuration. The dorsal and ventral rows consist of conventional ring-shaped contacts, whereas the central rows contain three separate directional contact segments. We first tested the “DBS off” condition, followed by the 10 possible monopolar electrode configurations (two conventional rings, six directional contacts, and two virtual rings) that were randomized *a priori* for each participant to preclude an ordering effect. We then measured the therapeutic window at a given DBS configuration, as described previously (Volkmann et al., 2006). The therapeutic window for each DBS configuration is defined as the ceiling value (i.e., 0.1 mA less than the current where the side effects were encountered) minus the floor (i.e., the current that provides significant improvement in cardinal signs). We delivered stimulation at the 50% midpoint of the therapeutic window at each DBS configuration. Walking occurred ~1 min after setting the stimulation amplitude within a given contact. We instructed participants for all trials to “walk at a speed that is most comfortable to you.” For all DBS configurations, individuals completed four 10-m walking trials. Walking speed and its independent constituent measures (i.e., step length and step time) were measured during the middle of the 10 m walk test (Graham et al., 2008; Hurt et al., 2018) by an instrumented 5.5 m walkway (Zeno, Protokinetics, Havertown, PA) and analyzed in Protokinetics Movement Analysis Software and custom scripts in MATLAB (Mathworks, Nantucket MA).

### Data Analysis

Gait data were grouped by contact number, and individual trials were averaged within each contact and ring in each participant. Walking speed is the product of step length and 1/(step time), which are independently controlled by individuals. We quantified the extent to which changes in walk speed related to changes in either step length or step frequency using a previously defined measure, the step length index (Hirasaki et al., 1999).

(1) $$\begin{array}{l} StepLengthIndex=\frac{\log\left(\frac{Step\;Length_{Max}}{Step\;Length_{Off}}\right)}{\log\left(\frac{CWS_{Max}}{CWS_{Off}}\right)}*100 \end{array}$$

A step length index of 50% implies an equal contribution of step length and step time for a given change in CWS. A step length index of 0% attributes the change in CWS completely to changes in step time, whereas an index of 100% would indicate that changes to CWS were attributed to changes in step length.

To measure differences in walking speeds across DBS configurations within individuals, we analyzed the maximum (i.e., fastest), minimum (i.e., slowest), and off DBS CWS with a one-way repeated measures ANOVA to assess the effect of DBS programming at point of care. *Post-hoc* tests with Bonferroni corrections assessed significant differences between conditions. The step length index was compared between the maximum measured CWS to both the off and minimum measured CWS condition between participants. A single sample *t*-test assessed whether the Step Length Index was significantly different than 50%, which would indicate that step length and step time equally contributed to the changes in walk speed. Intraclass correlation coefficient (ICC) estimates and 95% confidence intervals assessed the degree of reliability in walk speed across the four trials within each programming condition using SPSS statistical package version 25 (SPSS Inc., Chicago, IL) based on a two-way mixed effect model, absolute agreement. To test whether the number of trial repetitions might have impacted results, a Pearson chi-square test assessed the likelihood of the maximum CWS occurred in the first half or last half of the monopolar survey. Significance was set at *p* < 0.05 for all statistical tests.

## Results

**Figure 1:**
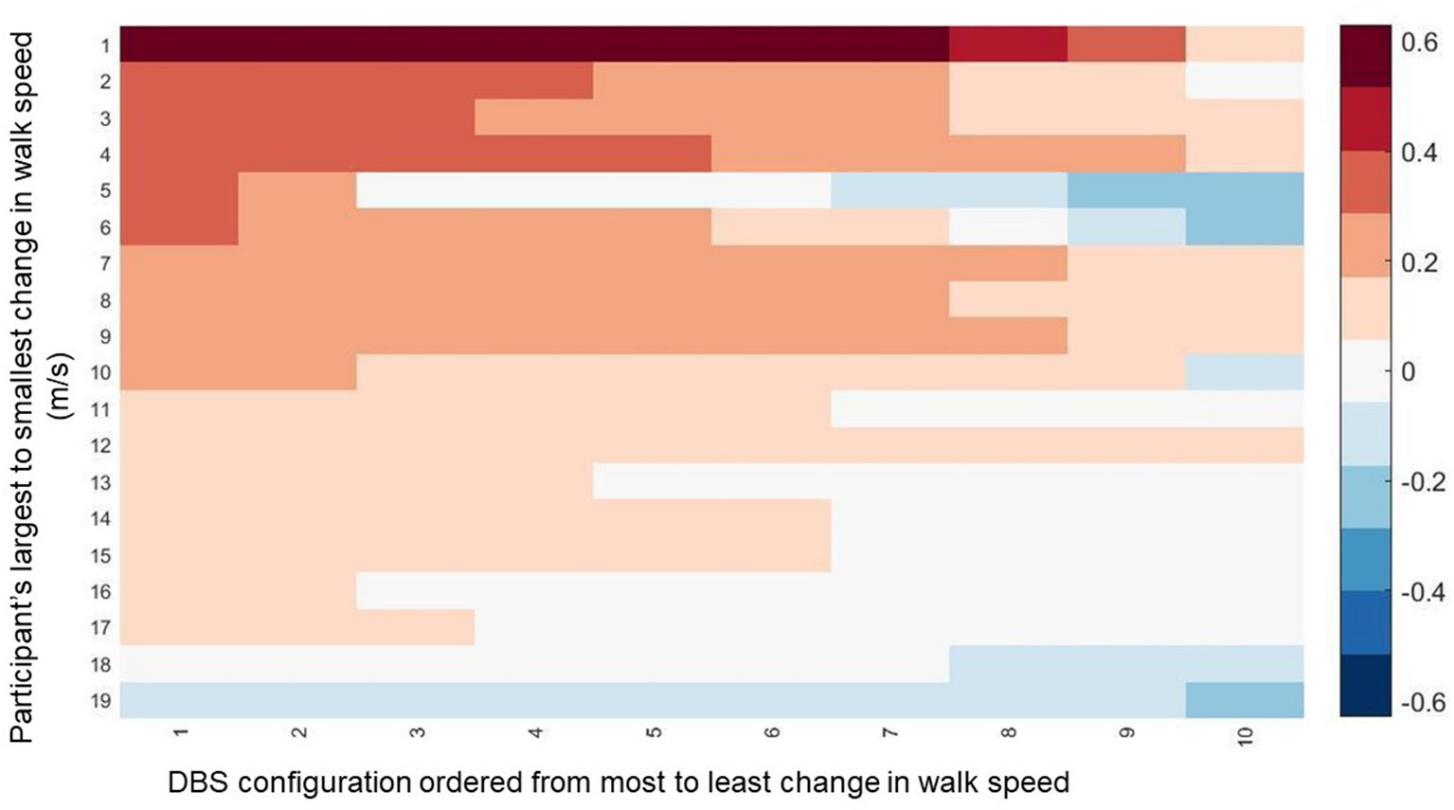
The change in walking speed is displayed across all DBS contact configurations for all participants. The data is ordered along the *y*-axis from those that experienced the biggest change in walking speed from Maximum CWS compared to DBS off to the smallest change. Along the *y*-axis, the data is ordered from the the DBS configuration for each participant that resulted in the greatest change in walking speed to the smallest change.

**Figure 2:**
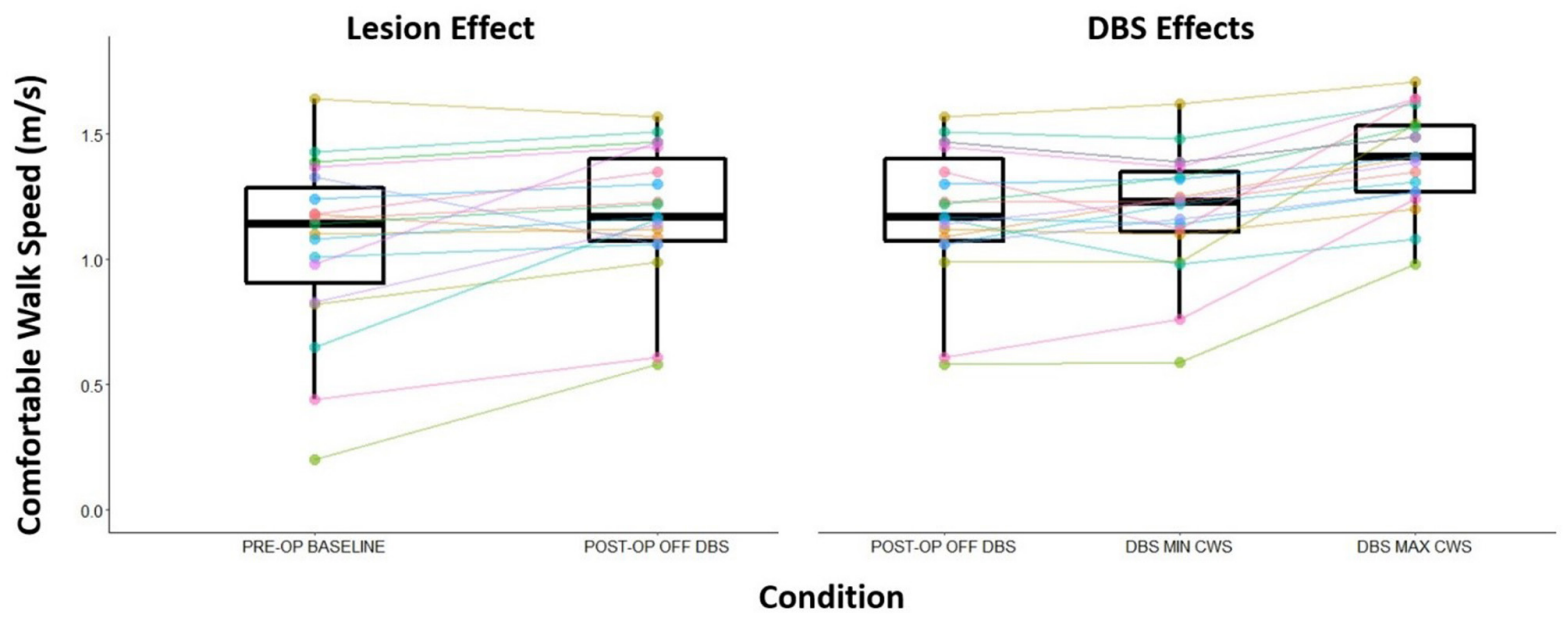
Change in CWS from the pre-operation baseline and post-operation OFF DBS (visualizing any lesion effect) and the effect of DBS to CWS. Post-operation OFF DBS trial to the maximum CWS recorded speed during DBS programming session to the minimum CWS recorded speed during the DBS programming session.

Walking speed differed substantially across directional and circular unilateral STN DBS configurations vs. both DBS off and pre-op baseline off, within and across participants (*p* < 0.001, respectively). Relative to DBS off conditions, a wide range of walking speeds were recorded for the given DBS configuration within patients and across patients (Figure 1). The maximum DBS CWS (1.38 ± 0.20 m/s) was significantly faster than the minimum DBS comfortable speed (1.18 ± 0.24 m/s), DBS off (1.18 ± 0.25 m/s), and pre-op baseline (1.06 ± 0.35 m/s, *p* < 0.001, respectively, Figure 2), whereas minimum comfortable DBS speed vs. DBS off did not differ significantly (*p* = 1.000). At least one directional DBS contact segment yielded a faster CWS than the best ring contact configuration in 63% of participants, whereas 18% of all DBS configurations yielded slower walking speeds vs. DBS off. Based on established clinical meaningful differences for changes in walk speed of individuals with PD (Hass et al., 2014), eight (42%) individuals experienced a medium effect (>0.14 m/s) and eight (42%) experienced a large effect (>0.22 m/s) on the change in CWS from DBS off to DBS maximum CWS. Differences within the DBS trials (maximum-minimum comfortable speed) for each individual showed that eight (42%) individuals experienced at least a medium clinically meaningful difference in the change in CWS during DBS testing. These clinically meaningful differences related primarily to increases in step length as opposed to step frequency [SLI index 66.7% ± 20.83 Off-Max CWS (*p* = 0.003), and 69.2% ±19.01 Off-Min CWS (*p* < 0.001)]. Step length significantly increased from the maximum DBS CWS (0.70 ± 0.08 m) compared to the minimum DBS CWS (0.63 ± 0.11 m) and off DBS conditions (0.63 ± 0.011, *p* < 0.001 for both comparisons). Step-length variability, a measure of the regularity of step length within each participant across trials, was significantly less for maximum DBS CWS (0.027 ± 0.010 m) than the minimum DBS CWS (0.036 ± 0.16 m, *p* = 0.009) and off DBS conditions (0.034 ± 0.017, *p* = 0.013). The trial-to-trial variance in CWS demonstrated reliability in this measure. ICC values for walking speed were all >0.949, suggesting excellent reliability (Table 2). Furthermore, 95% confidence intervals showed the lower limit of the interval was above 0.900 in 8/10 DBS trials and >0.883 in 2/10, suggesting excellent and good reliability, respectively. Finally, the maximum CWS occurred in the last half of the monopolar survey in 10 of the 19 participants (*p* = 0.392).

**Table 2:** Intraclass correlation coefficients for all 10 contacts tested and the off DBS case.

**Contact**	**1**	**2**	**3**	**4**	**5**	**6**	**7**	**8**	**9**	**10**	**Off**
ICC	0.975	0.949	0.964	0.964	0.955	0.959	0.96	0.96	0.959	0.971	0.971
95% C.I.	0.936	0.883	0.916	0.926	0.897	0.919	0.911	0.914	0.91	0.937	0.938
	0.99	0.979	0.985	0.985	0.982	0.982	0.984	0.983	0.982	0.988	0.988

## Discussion

Directional unilateral STN DBS elicits reliable, clinically meaningful changes in walking speed during device activation, both within and across participants. Specifically, a directional DBS electrode contact was associated with maximum CWS in 12 of 19 participants (63%), whereas circular (omnidirectional) stimulation maximally improved CWS in 7 of 19 (37%). Within individuals, gait speed displayed excellent reliability across repeated CWS trials within a given DBS configuration. Incorporation of quantitative gait assessments into DBS programming sessions could therefore be useful as an optimization tool during device programming, particularly in patients with gait dysfunction “off” medications. Furthermore, our gait findings build on prior work on interleaved stimulation and raise the hypothesis that more tailored directional stimulation fields could provide greater functional improvements in gait vs. circular stimulation in some patients (Weiss et al., 2013; Brosius et al., 2015).

A recent meta-analysis showed that bilateral DBS typically improves walking speed by an effect size of 0.6 taken from 27 studies (Roper et al., 2016). In the current investigation, we observed a 0.20 m/s difference between the minimum vs. the maximum CWS within individuals, which resulted in an effect size of 1.05. Here, optimized unilateral STN DBS resulted in the maximum improved walking speed by 0.32 ± 0.25 m/s vs. pre-op baseline, 0.20 ± 0.16 m/s from DBS off, and 0.21 ± 16 m/s using minimal CWS. All of these changes are large and clinically significant, based on prior validation studies in PD patients and a variety of health-related contexts. Also, we show that 18% of the tested contacts resulted in no change or even worsening of CWS vs. DBS off, similar to previous results (Kelly et al., 2010), suggesting that specific DBS settings can worsen gait and mobility when programming settings are not fully optimized. The use of directional DBS leads in the current study provides an opportunity to investigate the extent to which directional or traditional circular ring stimulation contribute to greater changes in CWS. While directional and circular DBS were both generally beneficial, a single directional contact, with its smaller surface area and higher impedance, acutely resulted in the greatest improvement in CWS in 63% of participants. Directional stimulation provides clinicians with greater flexibility to optimize stimulation parameters and could conceivably increase DBS efficacy for gait dysfunction in some patients.

We observed significant increases in step length to explain increases in CWS with different DBS stimulation configurations during monopolar programming sessions. For PD patients, compared to controls, smaller step length and similar cadences are observed even at matched walking speeds (Morris et al., 1996; Bayle et al., 2016; Peterson et al., 2020). Decreased step length may be related to increased inhibition from the basal ganglia to the thalamus and cortical motor structures, such as the primary motor cortex and the supplementary motor area (SMA) (Morris et al., 2005). Given the importance of these motor and premotor regions for scaling the size of movement parameters (Nachev et al., 2008), the reduced excitation of the SMA could lead to the hypokinetic gait pattern observed. With intentional control, increasing walk speed for individuals with PD results in an increase in step length and cadence, and in some cases, cadence may increase to a greater extent than step length (Peterson et al., 2020). However, prior studies show that PD therapies (i.e., medication and DBS) increase walk speed in individuals by increasing step lengths compared to the practically defined off condition (Johnsen et al., 2009; Navratilova et al., 2020). It is of interest that just the presence of stimulation did not alter changes to CWS or step length for the CWS minimum condition. However, our findings show that specific DBS settings can yield clinically significant improvements in CWS, mediated by improved scaling in step length, a fundamental subtask of locomotion.

CWS is a reliable measure of gait function during DBS programming and is consistent with non-DBS investigations in PD (Combs et al., 2014). Here we measured CWS as the clinician programmed the device to maximize benefit while minimizing side effects based primarily on the cardinal symptoms of rigidity, tremor, and bradykinesia. Ten unique DBS configurations yielded a distribution of walking speeds that, within each contact, were reliable across the four different repetitions, suggesting that a much smaller number of walking trials may be used during DBS programming with some confidence.

The clinically significant improvements to walk speed we observed with unilateral DBS are noteworthy. Improvements to gait function are important because they relate to overall mobility. However, safe community ambulation may involve other aspects of mobility not solely captured by gait function. Movement inhibition, compensatory step performance, and cognition (Hershey et al., 2008; Tabbal et al., 2008; Thevathasan et al., 2012; Mirabella et al., 2013; Mancini et al., 2018) are all important aspects related to safe community mobility (Peterson et al., 2016). For instance, suppressing planned responses (i.e., movement inhibition) to modify step parameters are important when walking in a complex or cluttered environment. The research is conflicting on the extent to which DBS can improve many of the aforementioned aspects of mobility and thus requires further study (Mirabella et al., 2013; St George et al., 2015; Mancini et al., 2018)

The current study has several limitations. Our sample size was relatively small, although our findings were consistent that walk speed is a reliable measure of function, and we saw significant changes with DBS. Walking speeds were measured acutely during initial device activation while participants were >12 h off PD medications. Future studies should better characterize longitudinal changes in and gait and mobility outcomes in response to directional stimulation. However, a recent study showed that changes in gait parameters from an initial DBS device activation persisted after 3 months (Navratilova et al., 2020). Additionally, these assessments were conducted “off” medications, and patients typically attempt to remain on medications in the home environment. Individuals performed the walk trials over 11 different conditions, which could lead to fatigue. Within the present study, participants rested while a clinician adjusted stimulation parameters. Programming took between 5 and 10 min, and individuals could rest longer if requested. Furthermore, the randomized order of the contacts tested for each participant showed that for 10 of 19 individuals, their nominal fastest speed occurred in the last half of the data collection, which statistically resulted in no difference in the likelihood that the fastest CWS occurred in the first or last half of the trials collected. This provided evidence that the results observed reflected no effect of fatigue upon repeated trials for the monopolar survey. Finally, during the programming sessions, we did not quantify how changes to stimulation parameters resulted in changes to other motor symptoms of PD besides gait, which may have provided more targeted insight on our results.

## Conclusion

Walking speed is a clinically meaningful, reliable measure of gait function that may be used to assess the acute effects DBS programming adjustments and maximize functional outcomes in patients with PD. Directional and circular unilateral subthalamic DBS in optimized configurations elicit acute and clinically significant improvements in gait dysfunction related to PD.

## Data Availability Statement

The raw data supporting the conclusions of this article will be made available by the authors, without undue reservation.

## Ethics Statement

The studies involving human participants were reviewed and approved by The Institutional Review Board of the University of Alabama at Birmingham. The patients/participants provided their written informed consent to participate in this study.

## Author Contributions

HW and CH: study design. CH, DK, MW, and CL: data collection. CH, DK, and CL: data analysis. CH, DK, MW, CL, HW, and BG: data interpretation and manuscript drafting and revising. All authors contributed to the article and approved the submitted version.

## Conflict of Interest

HW serves as a clinical scientific consultant for Medtronic and Boston Scientific. The remaining authors declare that the research was conducted in the absence of any commercial or financial relationships that could be construed as a potential conflict of interest.
